# Neutrophils are the source of IL-17 and RANKL in zymosan induced arthritis

**DOI:** 10.1186/ar3647

**Published:** 2012-02-09

**Authors:** Viktoriya I Milanova, Nina D Ivanovska, Petya A Dimitrova

**Affiliations:** 1 Department of Immunology, Institute of Microbiology, Bulgarian Academy of Sciences, Sofia, 1113, Bulgaria

## Background

Rheumatoid arthritis (RA) is a systemic inflammatory disease affecting cartilage and bone. Recently, much attention on the role of neutrophils in the pathology of RA has been paid. However, the capability of RA neutrophils from periphery and bone marrow (BM) to produce cytokines like IL-17 and IFN-γ has not been well understood. Our aim is to analyze neutrophil distribution in BM, blood and synovium and to elucidate IL-17, IL-4 and IFN-γ production and surface expression of RANKL on peripheral and synovial neutrophils during the progression of zymosan-induced arthritis (ZIA).

## Materials and methods

In the present study BALB/c and SCID mice were injected intra-articularly with zymosan. Cells from BM, periphery and synovium were collected at day 7 and day 30 of ZIA and the frequencies of Ly6G^+^CD11b^+ ^neutrophils and surface expression of RANKL and CD69 on them were evaluated by flow cytometry. In some experiments peripheral neutrophils were isolated at day 7 of ZIA, re-stimulated in vitro with zymosan in the presence or the absence of IL-17, then fixed, permeabilized and used for flow cytometry analyses of IL-17, IL-4 and IFN-γ intracellular levels and of surface RANKL expression. Apoptosis of cultured neutrophils was detected by annexin/propidium iodide kit. The ability of peripheral neutrophils to affect RANKL or IL-17-induced osteoclast differention of bone marrow precursors in vitro was evaluated after TRAP staining of cell co-cultures.

## Results

The development of inflammatory process in SCID mice after zymosan injection was related to increased frequencies of Ly6G^+^CD11b^+ ^neutrophils in periphery and synovium along with elevated IL-17 production in plasma and serum. We observed that arthritic neutrophils collected at day 7 of disease have higher IL-17, IL-4 and IFN-γ intracellular levels than healthy cells. Exogenous IL-17 increased the cytokine and RANKL expression on healthy and arthritic neutrophils in vitro. While neutrophils were able to inhibit RANKL-induced osteoclast differentiation, they increased the number of TRAP positive mature osteoclasts in the presence of IL-17.

## Conclusions

We suggest that Ly6G^+^CD11b^+ ^peripheral neutrophils that are positive for IL-17, IL-4, IFN-γ and RANKL can migrate to the synovium where they can affect inflammatory and destructive processes. Our study displays new aspect of the role of neutrophils in the pathology of RA and provides diverse ground for the development of novel therapeutic strategies.

